# Integrated omics for the identification of key functionalities in biological wastewater treatment microbial communities

**DOI:** 10.1111/1751-7915.12255

**Published:** 2015-02-12

**Authors:** Shaman Narayanasamy, Emilie E L Muller, Abdul R Sheik, Paul Wilmes

**Affiliations:** Luxembourg Centre for Systems Biomedicine, University of Luxembourg7 avenue des Hauts-Fourneaux, Esch-Sur-Alzette, L-4362, Luxembourg

## Abstract

Biological wastewater treatment plants harbour diverse and complex microbial communities which prominently serve as models for microbial ecology and mixed culture biotechnological processes. Integrated omic analyses (combined metagenomics, metatranscriptomics, metaproteomics and metabolomics) are currently gaining momentum towards providing enhanced understanding of community structure, function and dynamics *in situ* as well as offering the potential to discover novel biological functionalities within the framework of Eco-Systems Biology. The integration of information from genome to metabolome allows the establishment of associations between genetic potential and final phenotype, a feature not realizable by only considering single ‘omes’. Therefore, in our opinion, integrated omics will become the future standard for large-scale characterization of microbial consortia including those underpinning biological wastewater treatment processes. Systematically obtained time and space-resolved omic datasets will allow deconvolution of structure–function relationships by identifying key members and functions. Such knowledge will form the foundation for discovering novel genes on a much larger scale compared with previous efforts. In general, these insights will allow us to optimize microbial biotechnological processes either through better control of mixed culture processes or by use of more efficient enzymes in bioengineering applications.

## Biological wastewater treatment as a model system for Eco-Systems Biology

Biological wastewater treatment (BWWT), including the standard activated sludge process and other ancillary processes, relies on microbial community-driven remediation of municipal and industrial wastewater. Biological wastewater treatment plants host diverse and dynamic microbial communities possessing varied metabolic capabilities over changing environmental conditions, e.g. microorganisms accumulating various storage compounds of biotechnological importance. Given their structural and functional diversity, BWWT processes hold great potential for future sustainable production of various commodities from wastewater as well as from other mixed substrates (Muller *et al*., 2014; Sheik *et al*., 2014). Eco-Systems Biology is an integrative framework that includes systematic measurements, data integration, analysis, modelling, prediction, experimental validation (e.g. through targeted perturbations) and ultimately control of microbial ecosystems (Muller *et al*., 2013). This framework will aid in the understanding of BWWT processes by dissecting interactions among its constituent populations, their genes and the biotope, with the ultimate aim of maximizing biotechnological outcomes through various control strategies (Muller, Pinel *et al*., 2014,2014; Sheik *et al*., 2014).

**Fig 1:**
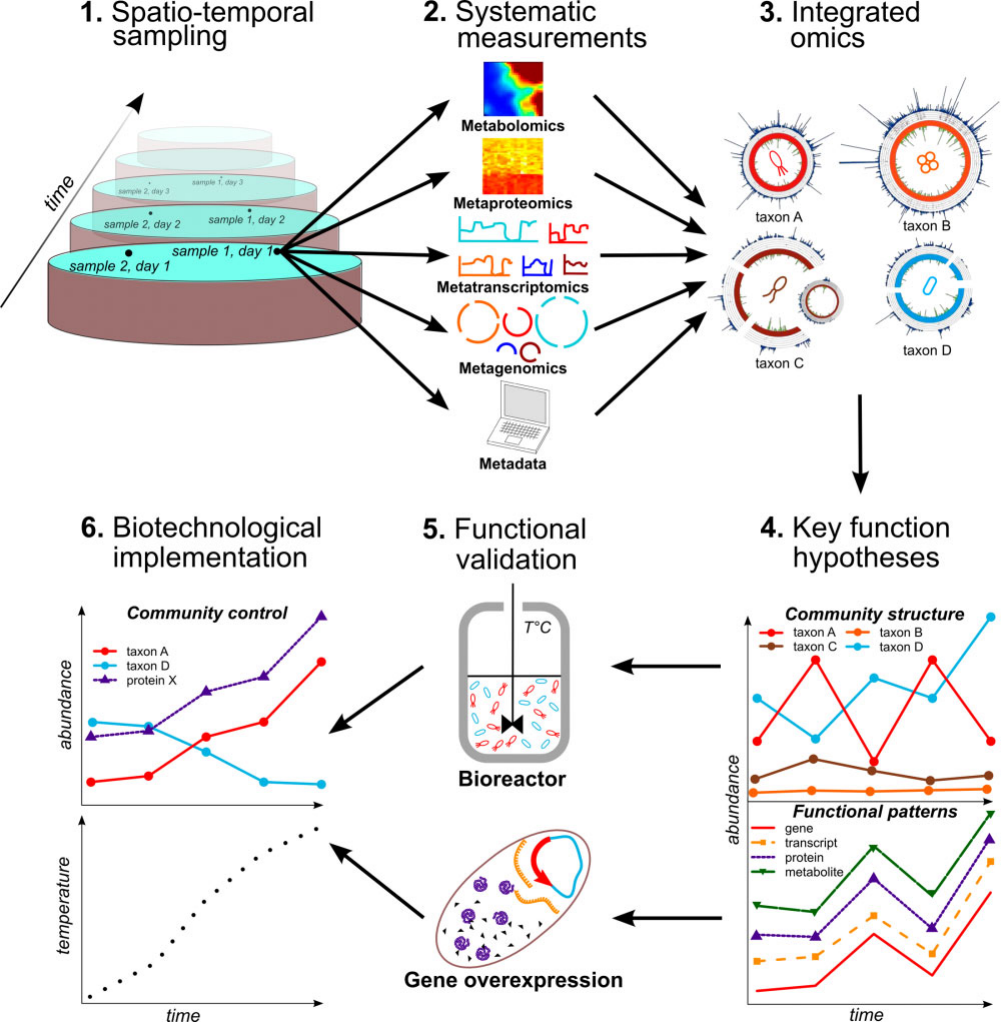
The path from large-scale integrated omics to hypothesis testing and biotechnological application in the context of biological wastewater treatment.

Biological wastewater treatment plants typically possess a relatively homogeneous environment (compared with most natural ecosystems) with well-defined physico-chemical boundaries and are widespread in developed and developing countries (Daims *et al*., 2006; Muller, Pinel *et al*., 2014; Sheik *et al*., 2014). Furthermore, contrary to other microbial habitats, e.g. the marine environment, acid mine drainage biofilms, the human gastrointestinal tract, etc., BWWT plants represent a convenient and virtually unlimited source of spatially and temporally resolved samples (Fig. 1; step 1). Physico-chemical parameters such as temperature, pH, oxygen and nutrient concentrations are routinely monitored and recorded, thereby facilitating hypothesis formulation and verification in rapid succession. This allows for example, the establishment of causal links between the influence of certain environmental parameters on microbial community structure and/or function derived from temporal sampling. Importantly, microbial consortia from BWWT plants are very amenable to experimental validation at differing scales, ranging from laboratory-scale bioreactors to full-scale plants (see section “From Eco-Systems Biology to biotechnology” below).

While being highly dynamic, microbial communities within BWWT plants maintain a medium to high range of diversity/complexity, thereby exhibiting a baseline stability over time such that there is temporal succession of repeatedly few quantitatively dominant populations (Albertsen *et al*., 2012; Zhang *et al*., 2012; Muller, Pinel *et al*., 2014; N. Pinel, pers. comm.). These characteristics reduce the complexity of downstream omic data analyses. In particular, given sufficient sequencing depth, current *de novo* metagenomic assemblers are highly effective for medium complexity communities, such as BWWT plant microbial communities (Segata *et al*., 2013; Muller, Pinel *et al*., 2014). Representative population-level genomic reconstructions can now be obtained for abundant community members (Albertsen *et al*., 2013; Muller, Pinel *et al*., 2014), and such genomic information is vital for the meaningful interpretation of additional functional omic data. Overall, BWWT plant microbial communities represent an important intermediary step/model between communities of lower diversity, e.g. acid mine drainage biofilms (Denef *et al*., 2010), and complex communities such as those from soil environments (Mocali and Benedetti, 2010), while retaining important hallmarks of both extremes including, for example, quantitative dominance of specific taxa (a characteristic of acid mine drainage biofilm communities), rapid stochastic environmental fluctuations (a characteristic of soil environments). Therefore, BWWT plant microbial communities exhibit important properties rendering them an ideal model for microbial ecology (Daims *et al*., 2006), and more specifically eco-systematic omic studies in line with a discovery-driven planning approach (Muller *et al*., 2013).

## Laboratory protocols, systematic measurements and *in silico* analyses

Mixed microbial communities, such as those present in BWWT plants, exhibit varying degrees of inter- and intra-sample heterogeneity, rendering standard (i.e. originally designed for pure isolate culture systems) biomolecular extractions protocols and computational analyses ineffective (Muller *et al*., 2013; Roume *et al*., 2013a,a). In our opinion, it is therefore absolutely essential to apply tailored and systematic approaches such as the biomolecular isolation protocol designed by Roume and colleagues (Roume *et al*., 2013a,a) to microbial communities. The protocol allows the sequential isolation of high-quality genomic deoxyribonucleic acid (DNA), ribonucleic acid (RNA), small RNA, proteins and metabolites from a single, undivided sample for subsequent systematic multi-omic measurements (Fig. 1, step 2). Importantly, this eliminates the need for subsampling the heterogeneous biomass and, therefore, reduces the noise arising from incongruous omics data in the subsequent downstream integration and analysis steps (Fig. 1, step 3; Muller *et al*., 2013; Roume *et al*., a,b).

Following standardized and systematized biomolecular isolations, multi-omic datasets are generated in addition to the physico-chemical parameters recorded at the time of sampling (Fig. 1; step 2). The multi-omic data are then subjected to bioinformatic pre-processing and analyses. Preliminary characterization of microbial communities can be facilitated either by high-throughput ribosomal RNA gene amplicon sequencing to determine broad community composition from shotgun metagenomic analyses to resolve the overall structure as well as the functional potential of the communities (Vanwonterghem *et al*., 2014). More importantly, hybrid *de novo* assemblies of metagenomic and metatranscriptomic reads promises higher quality compared with conventional *de novo* metagenomic assemblies due to the ability to reconstruct and resolve genomic complements of low abundance (i.e. low metagenomic coverage) yet highly active populations (i.e. high metatranscriptomic coverage for expressed genes; Muller, Pinel *et al*., 2014). Hybrid assemblies allow high-quality population-level genomic reconstructions after the application of binning/classification methods, such as those developed for a single sample (Laczny *et al*., 2014) or for spatio-temporally resolved samples (Albertsen *et al*., 2013; Alneberg *et al*., 2014; Nielsen *et al*., 2014). Furthermore, hybrid metagenomic and metatranscriptomic data assemblies allow the resolution of genetic variations with higher confidence through replication and highlights their potential relative importance, thereby allowing more detailed short-term evolutionary inferences regarding specific populations and while increasing sensitivity for downstream metaproteomic analysis (Muller, Pinel *et al*., 2014). Thus, the generation of metatranscriptomic and metaproteomic data is crucial to fully understand the functional capacity of microbial communities. Therefore, we believe that the integrated omic approach as elucidated by Muller and colleagues (Muller, Pinel *et al*., 2014), from systematic measurements to *in silico* analysis, is highly effective in: (i) minimizing errors by cancelling out noise and biases stemming from single omic analyses and (ii) optimizing/maximizing overall data usage.

Although high-throughput metagenomics and metatranscriptomics allow deep profiling of microbial communities at relatively low cost, existing sequence-based approaches do have some important limitations. Given the availability of omic technologies and their non-prohibitive costs (in particular for metagenomics and metatranscriptomics), fully integrated omic analyses should be applied routinely in the study of microbial consortia for greater effectiveness. For instance, despite this wealth of information, current metagenomic assemblies and analysis schemes, metagenomic (and metatranscriptomic) data resulting from the use of current short-read sequencing and assembly approaches do not allow the comprehensive resolution of microdiversity, e.g. genetic heterogeneity of microbial populations (Wilmes *et al*., 2009). Furthermore, RNAseq technologies are subject to biases stemming from the extensive, yet compulsory pre-processing steps (Lahens *et al*., 2014), thereby affecting the resulting metatranscriptomic data. On the other hand, chromatography and mass spectrometry-based metaproteomics and metabolomics currently remain limited in their profiling depth. While the situation for metaproteomics is rapidly improving (Hettich *et al*., 2012), community-wide metabolomic studies are still limited in their scope due to the poor detection/sensitivity of high-throughput metabolomic instruments and high dependency on a limited knowledgebase reflected in current metabolite databases. Overall, we anticipate significant technological advancements in all high-throughput measurement techniques particularly in the area of long-read sequencing, chromatography as well as mass spectrometry. Naturally, these technological improvements will be complemented by equally sophisticated *in silico* data processing and analysis methods, which in turn will allow integrated omics to provide comprehensive multi-level snapshots of microbial population structures and functions *in situ* (Fig. 1; step 3).

In our opinion, the real power of the integrated omics approach within the Eco-Systems Biology framework will stem from applying the approach to temporally and spatially resolved samples (Fig. 1, steps 1 to 4; Muller *et al*., 2013; Zarraonaindia *et al*., 2013). In combination with appropriate statistical and mathematical modelling methods, the deconvolution of the data will unveil unprecedented insights into the structure and function of microbial communities (Fig. 1; step 4; Muller *et al*., 2013; Segata *et al*., 2013; Zarraonaindia *et al*., 2013). Data mining, machine learning and/or modelling approaches will be useful for extracting features of interest, e.g. known and unknown populations/genes, and also to derive associations (or links) between desired features utilizing measures such as correlation, co-occurrence, mutual information and hyper-geometric overlap (Muller *et al*., 2013; Segata *et al*., 2013). Such associations may allow the prediction of gene functions using the concept of ‘guilt by association’ and interactions/dependencies between community members (Wolfe *et al*., 2009; Segata *et al*., 2013; Solomon *et al*., 2014). Biological wastewater treatment plants offer particularly exciting opportunities to link responses in community structure and function to fluctuating environmental conditions because of the relative ease of sampling and routine recording of metadata (Muller *et al*., 2013; Segata *et al*., 2013; Vanwonterghem *et al*., 2014). Systematic omic analyses of BWWT microbial communities may therefore uncover (i) the effect of physico-chemical parameters on the expression of specific genes or phenotypes and (ii) the linkage of unknown genes to specific metabolites as well as to both known and unknown community members. However, the derived associations will always be ‘mere’ hypotheses, which will require rigorous testing through targeted laboratory experiments (Fig. 1; step 5) and/or *in situ* perturbation experiments followed by additional omic measurements (Muller *et al*., 2013; Segata *et al*., 2013).

## Moving beyond associations and hypotheses

Although integrated omics-based approaches are highly effective for large-scale analysis and formulation of hypotheses (including within the context of BWWT plant communities), these efforts are limited due to current high-throughput measurement methods (see previous section) and the reliance on *a priori* knowledge for both taxonomical and functional inferences (Röling *et al*., 2010). Hence, there is a need to validate newly generated hypotheses using full-scale plants, customized laboratory-based experiments, such as batch cultures, bioreactors or pilot plants (Fig. 1; step 5) and/or single-cell methods. Hypotheses may be tested using additional integrated omic datasets generated from ancillary samples (e.g. Muller, Pinel *et al*, 2014) by using molecular biology techniques such as heterologous gene expression (e.g. Wexler *et al*., 2005; Maixner *et al*., 2008) or single-cell approaches using microautoradiography-fluorescent in situ hybridisation (MAR-FISH), nano-scale secondary-ion mass spectrometry (nanoSIMS) and/or Raman spectroscopy (e.g. Huang *et al*., 2007; Lechene *et al*., 2007; Musat *et al*., 2012). Such a combination of technologies can be used to test hypotheses regarding (i) community dynamics, (ii) gene expression patterns/interactions, (iii) metabolite abundances, (iv) effect of physico-chemical factors on distinct microbial species and functionalities, (v) gene function associations between any of these. Identified patterns may be subsequently formulated as cues and can be used as input to facilitate knowledge-driven control of different microbial community structures and/or functions (Fig. 1; step 6). Thus, large-scale integrated omic analyses of *in situ* biological samples (section “Laboratory protocols, systematic measurements and *in silico* analyses”), coupled to carefully controlled laboratory experiments, will allow the effective elucidation of novel functions within BWWT plant microbial communities with potential biotechnological applications.

## From Eco-Systems Biology to biotechnology

Knowledge of gene function, regulation and physiological potential derived from integrated omic data over different spatial and temporal scales holds great promise in harnessing the biotechnological potential of microbial consortia. In particular, advancements in integrated omics followed by hypothesis testing may generate new knowledge (Muller *et al*., 2013), which may for example be exploited in new approaches for the optimized production of biotechnologically relevant compounds under varying environmental conditions (Chen and Nielsen, 2013). The derived knowledge-base may further be used to fine-tune metabolic pathways at the transcriptional, translational and post-translational levels using the ever-expanding synthetic biology toolbox (Peralta-Yahya *et al*., 2012). Examples of possible future applications may include, for instance the bioengineering of fatty acid utilization and production for the production of biodiesel from ‘dirty’ mixed substrates, the engineering of different gene combinations for the production of various alcohols from mixed substrates (Lee *et al*., 2008) and the generation of hybrid processes by combining biological and chemical production steps resulting in new compounds that could serve as biofuels (Román-Leshkov *et al*., 2007). Through exploration of BWWT plant microbial consortia using integrated omics, we are therefore poised to unravel key functionalities, which will find applications in a whole range of different biotechnologies. In this context, integrated omics through facilitating direct linkages between genetic potential and final phenotype may become an essential tool in future bioprospecting. Therefore, in our opinion, integrated omics will become the standard means of analysing microbial consortia in the near future and will allow meta-omics to fulfil their promise for the comprehensive discovery of biotechnology-relevant microbial traits in natural consortia.

## Conflict of interest

None declared.

## References

[b1] Albertsen M, Hansen LBS, Saunders AM, Nielsen PH, Nielsen KL (2012). A metagenome of a full-scale microbial community carrying out enhanced biological phosphorus removal. ISME J.

[b2] Albertsen M, Hugenholtz P, Skarshewski A, Nielsen KL, Tyson GW, Nielsen PH (2013). Genome sequences of rare, uncultured bacteria obtained by differential coverage binning of multiple metagenomes. Nat Biotechnol.

[b3] Alneberg J, Bjarnason BS, de Bruijn I, Schirmer M, Quick J, Ijaz UZ (2014). Binning metagenomic contigs by coverage and composition. Nat Methods.

[b4] Chen Y, Nielsen J (2013). Advances in metabolic pathway and strain engineering paving the way for sustainable production of chemical building blocks. Curr Opin Biotechnol.

[b5] Daims H, Taylor MW, Wagner M (2006). Wastewater treatment: a model system for microbial ecology. Trends Biotechnol.

[b6] Denef VJ, Mueller RS, Banfield JF (2010). AMD biofilms: using model communities to study microbial evolution and ecological complexity in nature. ISME J.

[b7] Hettich RL, Sharma R, Chourey K, Giannone RJ (2012). Microbial metaproteomics: identifying the repertoire of proteins that microorganisms use to compete and cooperate in complex environmental communities. Curr Opin Microbiol.

[b8] Huang WE, Stoecker K, Griffiths R, Newbold L, Daims H, Whiteley AS, Wagner M (2007). Raman-FISH: combining stable-isotope Raman spectroscopy and fluorescence in situ hybridization for the single cell analysis of identity and function. Environ Microbiol.

[b9] Laczny CC, Pinel N, Vlassis N, Wilmes P (2014). Alignment-free visualization of metagenomic data by nonlinear dimension reduction. Sci Rep.

[b10] Lahens NF, Kavakli IH, Zhang R, Hayer K, Black MB, Dueck H (2014). IVT-seq reveals extreme bias in RNA-sequencing. Genome Biol.

[b11] Lechene CP, Luyten Y, McMahon G, Distel DL (2007). Quantitative imaging of nitrogen fixation by individual bacteria within animal cells. Science.

[b12] Lee SK, Chou H, Ham TS, Lee TS, Keasling JD (2008). Metabolic engineering of microorganisms for biofuels production: from bugs to synthetic biology to fuels. Curr Opin Biotechnol.

[b13] Maixner F, Wagner M, Lücker S, Pelletier E, Schmitz-esser S, Hace K (2008). Environmental genomics reveals a functional chlorite dismutase in the nitrite-oxidizing bacterium ‘Candidatus Nitrospira defluvii’. Environ Microbiol.

[b14] Mocali S, Benedetti A (2010). Exploring research frontiers in microbiology: the challenge of metagenomics in soil microbiology. Res Microbiol.

[b15] Muller EEL, Glaab E, May P, Vlassis N, Wilmes P (2013). Condensing the omics fog of microbial communities. Trends Microbiol.

[b16] Muller EEL, Sheik AR, Wilmes P (2014). Lipid-based biofuel production from wastewater. Curr Opin Biotechnol.

[b17] Muller EEL, Pinel N, Laczny CC, Hoopmann MR, Narayanasamy S, Lebrun LA (2014). Community integrated omics links the dominance of a microbial generalist to fine-tuned resource usage. Nat Commun.

[b18] Musat N, Foster R, Vagner T, Adam B, Kuypers MMM (2012). Detecting metabolic activities in single cells, with emphasis on nanoSIMS. FEMS Microbiol Rev.

[b19] Nielsen HB, Almeida M, Juncker AS, Rasmussen S, Li J, Sunagawa S (2014). Identification and assembly of genomes and genetic elements in complex metagenomic samples without using reference genomes. Nat Biotechnol.

[b20] Peralta-Yahya PP, Zhang F, del Cardayre SB, Keasling JD (2012). Microbial engineering for the production of advanced biofuels. Nature.

[b21] Román-Leshkov Y, Barrett CJ, Liu ZY, Dumesic JA (2007). Production of dimethylfuran for liquid fuels from biomass-derived carbohydrates. Nature.

[b22] Röling WFM, Ferrer M, Golyshin PN (2010). Systems approaches to microbial communities and their functioning. Curr Opin Biotechnol.

[b23] Roume H, Heintz-Buschart A, Muller EEL, Wilmes P (2013a). Sequential isolation of metabolites, RNA, DNA, and proteins from the same unique sample. Methods Enzymol.

[b24] Roume H, Muller EEL, Cordes T, Renaut J, Hiller K, Wilmes P (2013b). A biomolecular isolation framework for eco-systems biology. ISME J.

[b25] Segata N, Boernigen D, Tickle TL, Morgan XC, Garrett WS, Huttenhower C (2013). Computational meta'omics for microbial community studies. Mol Syst Biol.

[b26] Sheik AR, Muller EEL, Wilmes P (2014). A hundred years of activated sludge: time for a rethink. Front Microbiol.

[b27] Solomon KV, Haitjema CH, Thompson DA, O'Malley MA (2014). Extracting data from the muck: deriving biological insight from complex microbial communities and non-model organisms with next generation sequencing. Curr Opin Biotechnol.

[b28] Vanwonterghem I, Jensen PD, Ho DP, Batstone DJ, Tyson GW (2014). Linking microbial community structure, interactions and function in anaerobic digesters using new molecular techniques. Curr Opin Biotechnol.

[b29] Wexler M, Bond PL, Richardson DJ, Johnston AWB (2005). A wide host-range metagenomic library from a waste water treatment plant yields a novel alcohol/aldehyde dehydrogenase. Environ Microbiol.

[b30] Wilmes P, Simmons SL, Denef VJ, Banfield JF (2009). The dynamic genetic repertoire of microbial communities. FEMS Microbiol Rev.

[b31] Wolfe CJ, Kohane IS, Butte AJ (2005). Systematic survey reveals general applicability of ‘guilt-by-association’ within gene coexpression networks. BMC Bioinformatics.

[b32] Zarraonaindia I, Smith DP, Gilbert JA (2013). Beyond the genome: community-level analysis of the microbial world. Biol Philos.

[b33] Zhang T, Shao M-F, Ye L (2012). 454 pyrosequencing reveals bacterial diversity of activated sludge from 14 sewage treatment plants. ISME J.

